# Toward Intraoperative Margin Assessment Using a Deep Learning-Based Approach for Automatic Tumor Segmentation in Breast Lumpectomy Ultrasound Images

**DOI:** 10.3390/cancers15061652

**Published:** 2023-03-08

**Authors:** Dinusha Veluponnar, Lisanne L. de Boer, Freija Geldof, Lynn-Jade S. Jong, Marcos Da Silva Guimaraes, Marie-Jeanne T. F. D. Vrancken Peeters, Frederieke van Duijnhoven, Theo Ruers, Behdad Dashtbozorg

**Affiliations:** 1 Department of Surgery, Netherlands Cancer Institute, Plesmanlaan 121, 1066 CX Amsterdam, The Netherlands; 2 Department of Nanobiophysics, Faculty of Science and Technology, University of Twente, Drienerlolaan 5, 7522 NB Enschede, The Netherlands; 3 Department of Pathology, Netherlands Cancer Institute, Plesmanlaan 121, 1066 CX Amsterdam, The Netherlands

**Keywords:** ultrasound, breast cancer, deep learning, artificial intelligence, tumor segmentation, breast surgery, surgical margin

## Abstract

**Simple Summary:**

During breast-conserving surgeries, there is no accurate method available for evaluating the edges (margins) of breast cancer specimens to determine if the tumor has been removed completely. As a result, during the pathological examinations after 9% to 36% of breast-conserving surgeries, it is found that some tumor tissue is present on the margins of the removed tissue. This potentially leads to additional surgery or boost radiotherapy for these patients. Here, we evaluated the use of computer-aided delineation of tumor boundaries in ultrasound images in order to predict positive and close margins (distance from tumor to margin ≤ 2.0 mm). We found that our method has a sensitivity of 96% and a specificity of 76% for predicting positive and close margins in the pathology result. These promising results display that computer-aided US evaluation has great potential to be applied as a margin assessment tool during breast-conserving surgeries.

**Abstract:**

There is an unmet clinical need for an accurate, rapid and reliable tool for margin assessment during breast-conserving surgeries. Ultrasound offers the potential for a rapid, reproducible, and non-invasive method to assess margins. However, it is challenged by certain drawbacks, including a low signal-to-noise ratio, artifacts, and the need for experience with the acquirement and interpretation of images. A possible solution might be computer-aided ultrasound evaluation. In this study, we have developed new ensemble approaches for automated breast tumor segmentation. The ensemble approaches to predict positive and close margins (distance from tumor to margin ≤ 2.0 mm) in the ultrasound images were based on 8 pre-trained deep neural networks. The best optimum ensemble approach for segmentation attained a median Dice score of 0.88 on our data set. Furthermore, utilizing the segmentation results we were able to achieve a sensitivity of 96% and a specificity of 76% for predicting a close margin when compared to histology results. The promising results demonstrate the capability of AI-based ultrasound imaging as an intraoperative surgical margin assessment tool during breast-conserving surgery.

## 1. Introduction

Worldwide, breast cancer is the most prevalent type of cancer, with an estimation of 2.3 million new cases in 2020 [1]. Multiple trials have proven that breast-conserving surgery (BCS) followed by breast irradiation leads to optimal oncologic control, greater cosmetic outcome, and overall better quality of life compared to a mastectomy [2,3,4]. Therefore, breast-conserving therapy has become the preferred treatment method for breast cancer [3,4]. However, these superior outcomes are based on the achievement of tumor-free resection margins, considering the more than twofold increased risk for ipsilateral breast tumor recurrence in patients with positive resection margins [5,6]. The definition of what exactly constitutes a positive margin remains a worldwide debate. For invasive breast cancer, a positive margin is generally defined by tumor cells on the inked margin [7]. The reported rate of positive margins varies from 9% to 36% for invasive breast cancer [7]. In the case of positive margins, the patient may need additional surgery or boost radiotherapy, which both have a major impact on morbidity [8,9], cosmetic outcome [10,11,12], quality of life [13,14,15], and health care costs [16,17]. To reduce the number of patients with additional therapy and its associated risks and disadvantages, there is an unmet need for an accurate method for intraoperative evaluation of resection margins.

Breast ultrasound (BUS) is a quick, reproducible, non-invasive, inexpensive, highly feasible, and highly available method to assess margins. Multiple studies have found that the use of intraoperative BUS as a margin assessment tool reduces the number of positive margins in BCS patients [18,19,20,21,22,23,24]. Furthermore, Volders et al. have shown in a multicenter randomized controlled trial involving 134 patients, that ultrasound-guided BCS also leads to a better cosmetic outcome [25]. Despite these promising outcomes, several reasons have kept physicians from embracing BUS in their daily practice. One of the main reasons is the need for experience with the acquisition and interpretation of BUS images [26]. Sometimes the interpretation of BUS images is even more difficult due to a low signal-to-noise ratio and artifacts [26]. To obviate these shortcomings, efficient computer-aided BUS evaluation methods are needed to realize a fast, accurate, and operator-independent method for margin assessment. One of the principal steps in computer-aided BUS evaluation for margin assessment is the delineation of the lesion boundary based on certain attributes [27]. This process step is more commonly known as image segmentation.

In the domain of breast cancer diagnosis, many studies have been conducted regarding BUS segmentation [28]. For BUS segmentation, several traditional methods have been used, including thresholding algorithms [29,30], region growing methods [31], watershed methods [32,33], graph-based methods [34,35], and deformable models [36,37]. Segmentation methods based on machine learning include clustering [38] and support vector machines [39]. Recently, deep artificial neural networks have shown application in various medical image segmentation tasks. Several studies have found that the use of deep convolutional neural networks (CNNs) outperforms the earlier described classical approaches and conventional machine learning methods when it comes to the segmentation of BUS images [40,41,42,43,44]. However, many of these studies report promising segmentation results that are based on CNN models trained and tested on BUS data sets with both benign and malignant masses [40,41,42]. There is a lack of data regarding segmentation performance on data sets purely containing malignant tumors. This is an important issue since malignant tumors are much more difficult to segment due to irregular contours, speculation, and angular margins, while benign tumors are hyperechogenic and often have a smooth, ellipsoid shape [45]. On top of that, many studies report training and testing of BUS segmentation methods on relatively small and nonpublic data sets with distinct properties. These factors limit the application to a new data set. The few studies that do report segmentation results on purely malignant images, often report lower performance results [43,44,46]. For instance, Badawy et al. reported that the best-performing network in their study, the DeepLabV3+ ResNet50 network, had a mean boundary F1-score of 83% on a data set of 200 benign images, while it had a mean boundary F1 score of 60% on a data set of 200 malignant images [44].

In light of the aforementioned limitation, Gómez-Flores et al. have evaluated and published 8 artificial neural network segmentation models pre-trained on an extensive data set of 3061 BUS images, acquired from seven different US devices [47]. The goal of their study was to make these models available for other research groups in order to reproduce and improve the segmentation performance on new data sets. They included the following individual models; (1) the fully convolutional network based on AlexNet, (2) the U-Net, the SegNet architectures based on (3) VGG16 and (4) VGG19, the DeepLabV3+ architectures based on (5) ResNet18, (6) ResNet50, (7) MobileNet-V2, and (8) Xception. They evaluated their models using, amongst others, the median Dice Similarity Coefficient (DSC) [47]. DSCs were reported for BUS images of benign tumors and BUS images of malignant tumors separately. The highest median DSC of an individual network for delineating benign tumors was 0.92 (ResNet50) [47]. When it came to segmenting malignant tumors, all networks performed slightly less accurately with the highest median DSC being 0.88 (ResNet 50).

These results are very promising, and raise the question of whether these models could be used in practice for margin assessment of breast cancer specimens. There are several factors that should be investigated before these models can be broadly applied for routine intra-operative resection margin assessment on specimens. The first one is that the algorithms may perform worse when tested on US images acquired with different US devices compared to the US images in the training set [28,40]. Even though the authors used 7 different US devices for data acquisition, the different transducer characteristics or settings in a new data set could hamper the performance. Furthermore, the used BUS images were acquired during routine diagnostic breast studies. These images are captured from the outside of the breast and cover a larger part of the breast compared to BUS images of BCS specimens. Specimen images display less contextual information of the surrounding healthy tissue, and the tumor boundaries are more superficial which might cause the segmentation to be more challenging.

In order to make BUS segmentation clinically applicable for supporting margin assessment, we have taken the opportunity to investigate how we could optimize the tumor segmentation and margin assessment performance of the pre-trained well-known artificial neural networks when applied on a new data set of BUS images acquired on BCS specimens. To our best knowledge, no studies have been conducted yet to investigate this. In this study, we aim to determine the diagnostic accuracy of ensemble learning techniques for automated BUS segmentation based on deep learning networks for predicting close margins ≤ 2.0 mm after validating and improving their segmentation performance. For this purpose, we first acquired a BUS image data set of only specimens containing biopsy-proven malignant tumors. Then we used all pre-trained neural networks to segment the tumor in the BUS images. These segmentation maps formed the basis for the main contributions of this study, which are listed as follows:

(1) Evaluation of the segmentation performance of eight individual pre-trained artificial neural network models on an external data set, applied to a new data set of US images acquired on BCS specimens with invasive carcinoma lesions (BCS-US data set).
(2) Evaluation of the influence of an ensemble framework by combining multiple networks on the segmentation performance. Various methods for this ensemble approach were used, including different forms of voting, and weighted average.
(3) Optimization of the best ensemble approach, in order to predict margin status with the highest accuracy.

## 2. Materials and Methods

### 2.1. Data Acquisition

**Figure 1:**
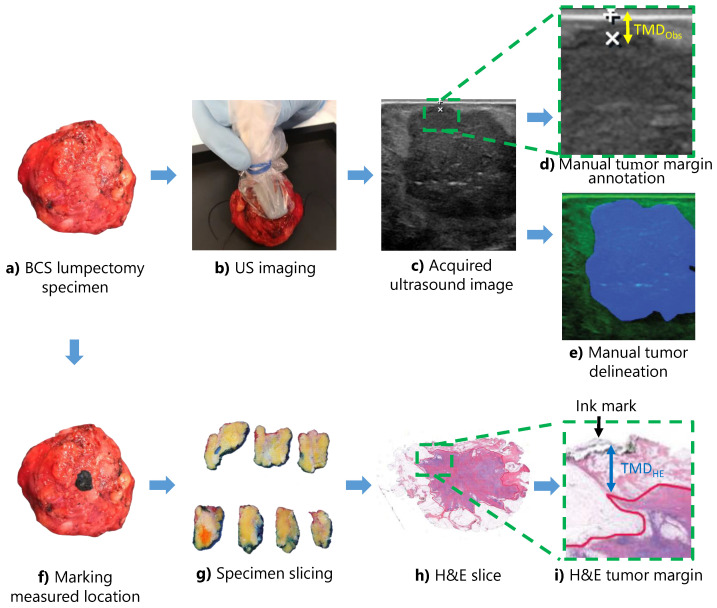
Overview of the method for data acquisition on lumpectomy specimens. An US image (**b**,**c**) was captured on a tissue location on the lumpectomy specimen (**a**) where the shortest margin ($TMD_{Obs}$) was present (**c**). In this image, the distance was measured and the location of this distance was marked with the ultrasound device annotation tool (white crosses in (**d**)). On the actual margin of the specimen, this location was marked with black ink to enable correlation with histopathology (**f**). After data acquisition, the BCS specimen was further processed according to standard protocol, including slicing of the tissue (**g**). The microscopic H&E slices were annotated by a pathologist and the $TMD_{HE}$ was obtained by measuring the minimum TMD from the marked margin region to the tumor in the H&E slice (**h**,**i**). Additionally, the tumor lesion in the BUS image was manually delineated by two independent observers (blue shade in (**e**)).

An overview of the data acquisition method can be found in Figure 1. BCS-US images were acquired from breast cancer specimens of patients with invasive carcinoma or invasive carcinoma combined with DCIS or LCIS, who had undergone breast-conserving surgery at the Netherlands Cancer Institute (Antoni van Leeuwenhoek hospital). According to the medical research involving human subjects act, no written consent was required. The images were collected using the Philips CX50 ultrasound device combined with a Philips L15-7io high-frequency transducer (Philips Research, Eindhoven, The Netherlands). In order to get more accurate information about the specimen margin and the superficial tumor boundary, we used the maximum frequency of 15 MHz (highest superficial resolution). In this way we could obtain an imaging depth of approximately 3 cm. Per specimen, 1 or 2 images were captured at locations with the shortest radial distance from the edge of the tumor to the margin, the tumor-margin distance (TMD), based on visual inspection of the US image. The exact visual distance at these locations in the US image was measured using the caliper function of the US device. Hereafter, this distance will be referred to as the $TMD_{Obs}$. Each measured tissue location was marked with black pathology ink. Afterward, standard pathology processing was applied. The pathology H&E sections of all measurement locations were digitized and examined by an experienced pathologist, who annotated all tumor tissue present in the images. The minimum TMD was determined in all H&E sections from the margin region marked with black ink to the tumor (mm). From now on this distance will be referred to as the $TMD_{HE}$.

### 2.2. Data Labeling

In order to compile a pixel-level ground truth map for each BUS image, the tumor boundaries were manually outlined by two independent experts. Based on these manual annotations, all pixels within the boundaries were considered to constitute the tumor region, while the remaining pixels were considered to be healthy tissue. Then a majority voting method was applied, where a pixel was labeled as tumorous tissue (1) if at least one observer annotated it as such, otherwise it was labeled as healthy (0). In this way, we generated binary, ground truth images.

### 2.3. Tumor Segmentation Methodology

The pre-trained deep neural network models developed by Gomez-Flores et al. [47] were used for tumor segmentation in the BCS-US data set. The gray-scale BCS-US images were cropped and rescaled to a size of 128×128 pixels, replicated two times and concatenated, to make them suitable for an input layer of 128×128×3 for the neural network models. The final output layer of the networks assigns a tumor probability value to each pixel. These probability values were values between 0 and 1. When multiple networks were combined, as described below, the probability values were summed up and normalized to a value between 0 and 1. Eventually, a pixel was classified as tumorous tissue if the probability value was greater than a certain threshold. Otherwise, it was classified as healthy tissue.

### 2.4. Ensemble Learning Framework

**Figure 2:**
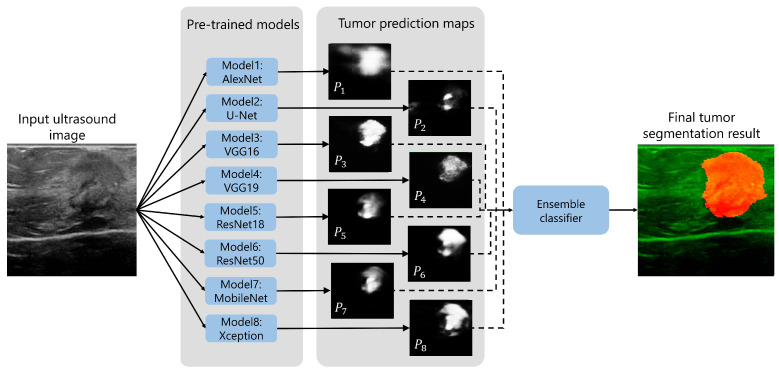
The described framework of ensemble learning for tumor segmentation in BCS-US images, using 8 models pre-trained on a public breast US data. In the output image, healthy tissue is highlighted in green, and tumor tissue is highlighted in red.

First, the segmentation performances of all individual models were tested on all images of the BCS-US data set. Subsequently, several ensemble approaches were tested. Figure 2 illustrates the general pipeline for using these ensemble learning methods.

#### 2.4.1. General Voting Method

**Figure 3:**
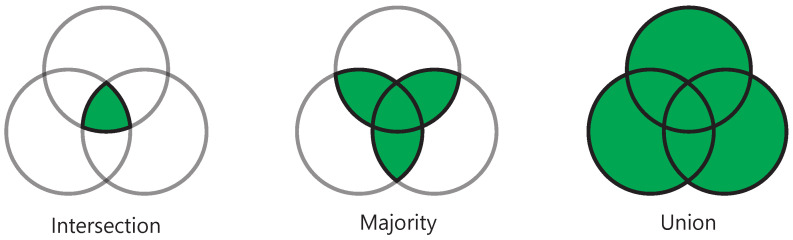
A schematic overview illustrating the 3 voting approaches. Each circle represents a tumor segmentation result by one model (3 models in total). Each voting method generates a white area, which is the predicted healthy area, and a green-shaded area, which is the predicted tumorous area.

Each network had an equal vote on whether a pixel belonged to the class of tumorous tissue or not. From all votes, three different predictions were formed based on a different selection of votes, as illustrated in Figure 3. The intersection selection, predicted a pixel to be tumorous if all models predicted it as such. The majority selection, predicted a pixel to be tumorous if 50% or more of all models predicted it as such. Lastly, the union selection predicted a pixel to be tumorous if any model predicted it as such.

#### 2.4.2. Elective Voting Method

An elective voting method was used where all possible combinations of 2 to 8 networks were tested using the union, majority and intersection voting approaches as described above.

#### 2.4.3. Weighted Average Method

The previously described methods were focused on combining the segmentation maps of the models using different votes. This meant that some models had an impact on the final segmentation map, and others did not, depending on the voting system. To balance the influence of all models, we combined all models while assigning a different level of impact (weight) to each model on the final segmentation map. In this approach, we combined the results of the models using different probability thresholds and different weights. In this way, we conducted an extensive grid search to find the optimum network-threshold-weight combination (NTWC) yielding the highest mean segmentation performance over all BCS-US images. For this purpose, weights are assigned to the probability maps of all individual networks, which are afterward summed up and normalized by the total sum of weights. In order to obtain the final segmentation results, a threshold value was applied to the final probability map. The optimal weights and threshold values are obtained using a grid search optimization technique. The grid search algorithm finds the optimal parameters by searching exhaustively through the range of a manually specified parameter which maximizes the output performance. For parameter optimization, weights were varied over a preset range. One network would be assigned no weight (weight of ’0’), the other seven networks would have a weight between 0 and 0.98, with an equal step size of 0.14 (1/7 = 0.14). Furthermore, different threshold values between 0 and 1 were used for the networks, with a step size of 0.02.

For further investigation on the impact of the threshold value and the weight of each model on segmentation results, the NTWCs with a median segmentation performance (Dice score) greater than a selected cut-off point of 0.85 were selected. This particular cut-off point was the minimum segmentation peformance of the individual networks investigated by Gomez-Flores et al. The average weight of each network among this selection was determined. Additionally, the frequency of each threshold value was evaluated. The best NTWC for BCS-US segmentation was selected by choosing the most frequent threshold and weight combination based on the average weight results of the best-performing NTWCs.

### 2.5. Tumor Margin Distance Prediction

In the following step, we investigated how we could use an ensemble approach for BCS-US segmentation to accurately predict the tumor-margin distance. For this, we first repeated the parameter optimization for achieving the lowest margin error. The model combination with the highest sensitivity and specificity for detecting a close margin using the $TMD_{HE}$ as ground truth, was selected as the final segmentation approach. After applying the final segmentation method on the entire BCS-US images, the extracted tumor masks are used for predicting the surgical tumor-margin distance. The predicted tumor-margin distance, $TMD_{Pred}$, is defined as the shortest distance between the surface of the lumpectomy (top of US image) to the detected tumor mask.

### 2.6. Performance Evaluation

#### 2.6.1. Tumor Segmentation

The segmentation performance was evaluated using the median DSC of all tested US images. The DSC measures the level of similarity between the predicted segmented lesion and the extracted lesion by the human observer (ground truth map). The DSC score ranges from 0 indicating no overlap at all, to 1 indicating perfect overlap.

A One-way Analysis of Variance (ANOVA) (Kruskal-Wallis test), a rank-based nonparametric test, was performed to determine whether there were statistically significant differences between the segmentation performance of all individual networks. In this test, a *p*-value ≤0.05 is considered as significant. Additionally, a Kruskal-Wallis test was performed to find any significant differences between the segmentation performance of the different ensemble approaches.

Furthermore, to evaluate the robustness of the final tumor segmentation approach, it was tested on three randomly divided subsets of the existing data. This is to evaluate whether the segmentation performance would be comparable for the whole patient population, or if there was a lot of influence by outliers. Again, a Kruskal-Wallis test was performed to determine if there were statistically significant differences between the performance of the final segmentation approach on each of the three data subsets.

#### 2.6.2. Margin Assessment

The predicted $TMD_{Pred}$ was compared to two types of ground truth; (1) margin calculated based on expert US annotation $TMD_{Obs}$, and (2) margin obtained from microscopic histology images $TMD_{HE}$.

Additionally, multiple metrics were measured to evaluate the distance error, including the mean absolute error (MAE), the normalized root-mean-square-error (NRMSE) and the Pearson correlation coefficient (PCC).

A two-sample *t*-test was performed to test whether there was a significant difference in all metrics between the $TMD_{Pred}$ and both ground truths (*p* < 0.05).

Furthermore, the sensitivity and specificity of diagnosing a close margin were determined, by comparing the predicted results with human annotations and the margin status documented in the histology report. For this study, we defined a close margin as a TMD ≤ 2.0 mm.

## 3. Results

### 3.1. Tumor and Patient Characteristics

In total, 109 BUS images of malignant tumors were acquired from 86 BCS specimens originating from 86 different patients. The median age of the patient population was 57 years (SD = 12.4). As far as the pathological diagnosis of the patients, 35 patients (41%) had an invasive carcinoma of no special type, 43 patients (50%) had an invasive carcinoma of no special type combined with DCIS, 3 patients (3%) had an invasive lobular carcinoma, and 5 patients (6%) had an invasive lobular carcinoma combined with lobular carcinoma in situ. Of all included patients, 16 patients (19%) had received neoadjuvant chemotherapy and 14 patients (16%) had received neoadjuvant hormonal therapy. An overview of the tumor and patient characteristics can be found in Table 1.

### 3.2. Tumor Segmentation

#### 3.2.1. Individual Models

**Figure 4:**
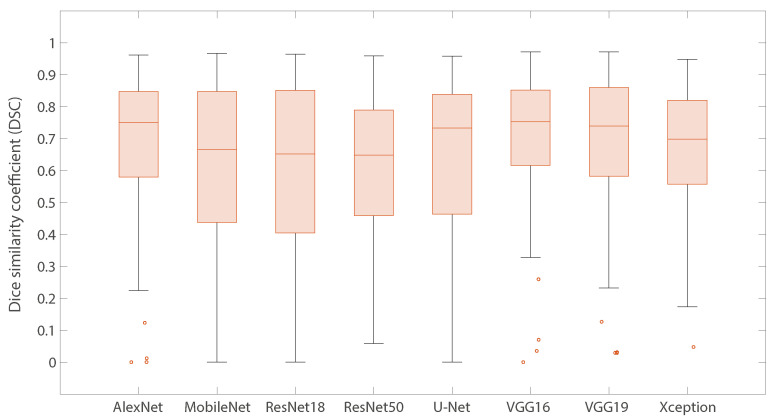
Boxplots demonstrating the median, minimum, maximum, first quartile, and third quartile values of the DSC scores for all individual models.

In Figure 4, the boxes display the median, minimum, maximum, first quartile and third quartile values of the DSCs of all individual models. As stated earlier the DSC measures the level of similarity between the predicted segmented lesion and the extracted lesion by the human expert (ground truth map). Notably, two networks attained the highest median DSC on the BCS-US data set: AlexNet (0.75, IQR: 0.27) and VGG16 (0.75, IQR: 0.23), while the ResNet50 network attained the lowest median DSC (0.65, IQR: 0.33). A Kruskal-Wallis test showed no significant differences between the segmentation performance of all individual networks.

#### 3.2.2. Ensemble Learning (Models Combinations)

**Figure 5:**
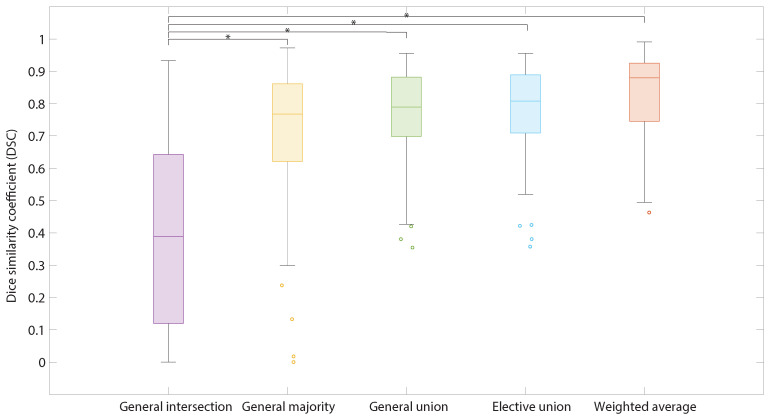
Boxplots demonstrating the segmentation performance of different ensemble approaches. The lines with an asterisk indicate a *p*-value < 0.05.

The boxes in Figure 5 exhibit the median DSC when applying the described ensemble approaches in Section 2.4. The first three boxes from left to right display the median DSC for general intersection (0.39, IQR: 0.52), the general majority (0.77, IQR: 0.24), and general union voting (0.79, IQR: 0.18), respectively. It is apparent that the general intersection method has the worst segmentation performance, with the lowest median DSC and the highest variability in DSC scores among individual BCS-US images. There is an increasing trend in the median DSC when switching from general intersection, to general majority to general union voting. The fourth box displays the DSC scores for the elective union method (0.81, IQR: 0.18), which had the highest median DSC among the elective and general voting methods. The fifth box visualizes the DSCs of the optimum weighting average combinations technique with the highest median DSC score. The weighted average approach achieved a median DSC of 0.88 (IQR: 0.18). A Kruskal-Wallis test showed there were statistically significant differences *p*-value < 0.05) in the performance of the general intersection voting method compared to all other depicted ensemble approaches.

#### 3.2.3. Weighting Average Parameters Optimization

An exhaustive grid search method was used to obtain optimal parameters (weights and threshold) for the weighted average ensemble learning approach. The optimization was performed two times, once for achieving the maximum Dice score and the second time to maximize the sensitivity of margin assessment.

Highest Dice Similarity Coefficient

**Figure 6:**
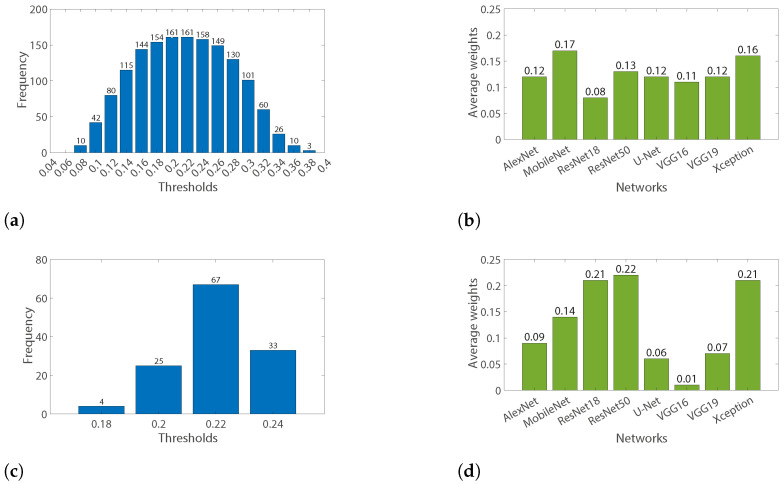
(**a**) Frequency of thresholds among NTWCs with a high segmentation performance, (**b**) Average weight of each network in all NTWCs with a high segmentation performance, (**c**) Frequency of thresholds among NTWCs with a high margin assessment performance, (**d**) Average weight of each network in all NTWCs with a high margin assessment performance.

The optimal parameters (threshold value and average weight factors per network) for the weighted average method are obtained from the best combinations with a median DSC of higher than 0.85 (Figure 6). Figure 6a displays the frequency of thresholds among the selected group of model combinations, according to which a threshold of 0.20 or 0.22 seems most optimal. The average weights for each network among the top-performing combinations are displayed in Figure 6b.

Highest Margin Assessment

The parameter optimization was done in a manner to achieve the highest margin assessment performance for detecting a close margin. The NTWCs capable of achieving a sensitivity > 95% and a specificity > 75% compared to the $TMD_{HE}$ as ground truth were further selected and analyzed. Figure 6c displays the frequency of thresholds among this group, according to which a threshold of 0.22 seems most optimal, similar to the NTWCs with a high segmentation performance. The mean weight of each network among this group is displayed in Figure 6d.

The final selected weights and threshold values after the parameter optimization based on the aforementioned criteria are shown in Table 2.

Furthermore, the segmentation performance of this final segmentation method with optimal parameters was compared on three randomly selected subsets of BCS-US images with equal sizes. The median DSC scores of these three subsets are shown in Table 3. A Kruskall-Wallis test revealed a *p*-value of 0.47, meaning there is no significant difference between the medians of all three data subsets. This demonstrates the robustness of the proposed framework.

### 3.3. Margin Assessment

The predicted margins ($TMD_{Pred}$) obtained using the optimized weighted average ensemble learning method are compared with margins extracted from the images annotated by the human experts ($TMD_{Obs}$) and margins based on histology images ($TMD_{HE}$). The results are shown in Table 4. When comparing the $TMD_{Pred}$ to the $TMD_{Obs}$, the MAE was 0.83 mm, the NRMSE was 0.21, and the PCC was 0.70. A close margin (≤2.0 mm) could be predicted with a sensitivity of 95% and a specificity of 57%. When comparing the $TMD_{Pred}$ to the $TMD_{HE}$, the MAE was 0.57 mm, the NRMSE was 0.16, and the PCC was 0.72. In this scenario, a close margin could be predicted with a sensitivity of 96% and a specificity of 76%.

**Figure 7:**
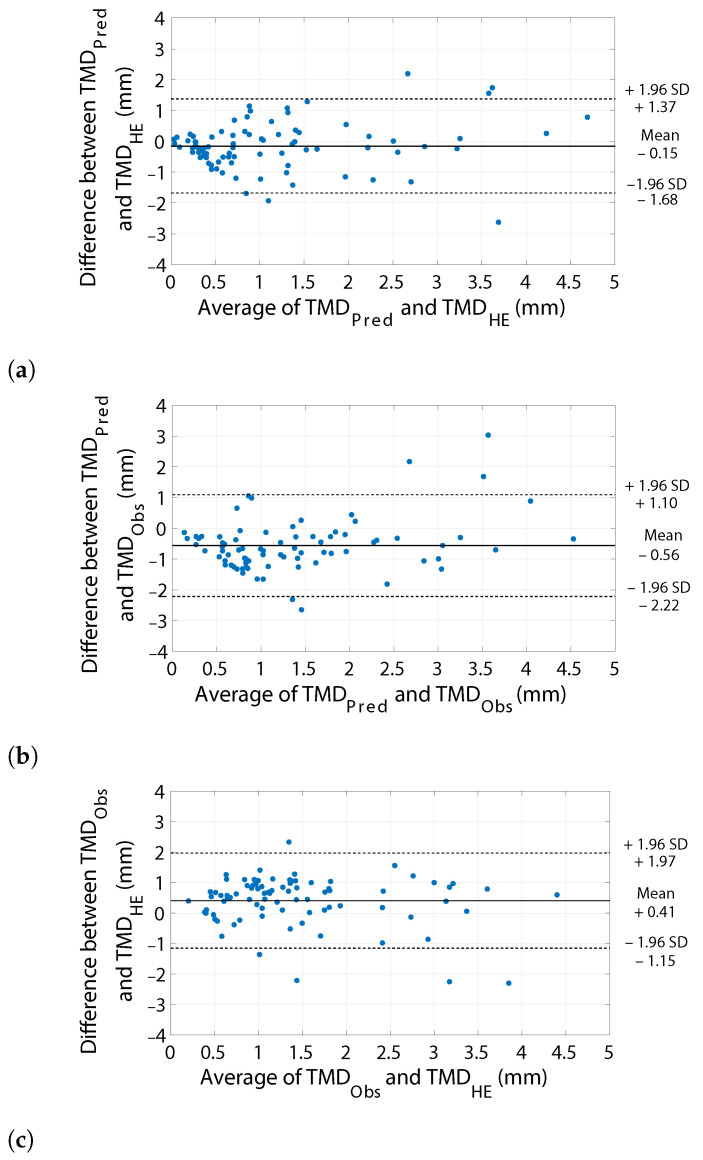
Bland-Altman plots of the agreement between (**a**) the $TMD_{Pred}$ and the $TMD_{HE}$ as a reference value, (**b**) the $TMD_{Pred}$ and the $TMD_{Obs}$ as a reference value, (**c**) the $TMD_{Obs}$ and the $TMD_{HE}$ as a reference value. Solid line: mean difference; dashed lines: 95% upper and lower limits of agreement.

The Bland-Altman plots in Figure 7 visualize the difference and agreement between different tumor margin measurement methods by considering one measurement method as predicted value and the other one as reference (ground truth) value. In these plots, the agreement between the compared TMDs was reported as a bias (average difference) together with the upper and lower limits of the 95% confidence interval for this average difference.

The agreement between the $TMD_{Pred}$ and the $TMD_{HE}$ can be observed in Figure 7a. There is a mean bias of −0.15 mm (95% CI: −1.68, 1.37), meaning both TMDs are quite similar. Correspondingly, a two-sample *t*-test showed no difference in the mean of both TMDs at the 5% significance level (Table 4). The mean bias refers to the average difference in measurements between two measurement methods, which could have a positive or negative value. Positive values indicate general underestimation of the compared method, while negative values indicate general overestimation of the compared method. This is different from the the MAE, which refers to the average of all absolute errors, and always has a positive value.

The agreement between the $TMD_{Pred}$ and the $TMD_{Obs}$ can be observed in Figure 7b. It shows a mean bias of −0.56 mm (95% CI: −2.22, 1.10), and the difference between the mean of both TMDs is significant at the 5% significance level according to a two-sample *t*-test (Table 4).

When comparing the $TMD_{Obs}$ to the $TMD_{HE}$, the MAE was 0.73 mm, the NRMSE was 0.19, and the PCC was 0.69 (Table 4). The agreement between the $TMD_{Obs}$ and the $TMD_{HE}$ can be observed in Figure 7c. It shows a mean bias of 0.41 mm (95% CI: −1.15, 1.97). Similarly, a two-sample *t*-test showed a significant difference in the mean of both TMDs at the 5% significance level (Table 4).

**Figure 8:**
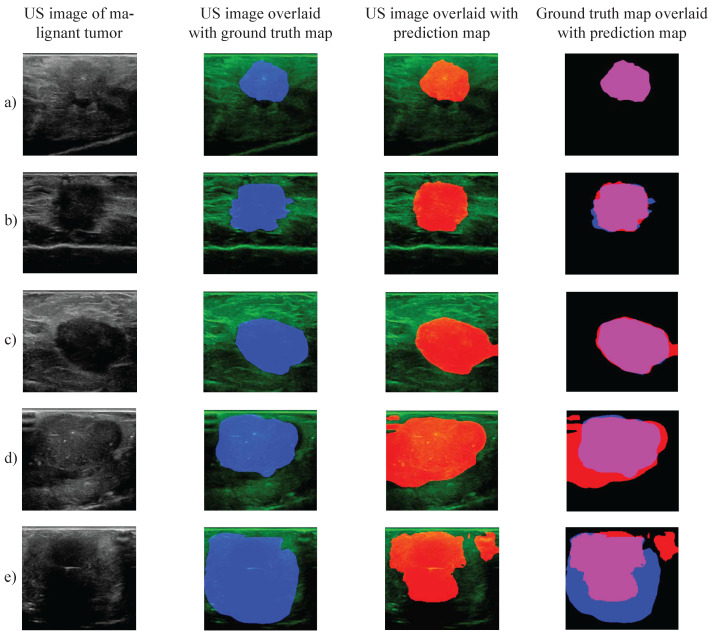
5 examples of BCS-US segmentation of different invasive carcinoma lesions. (**a**–**c**) images of lesions with high DSC scores and accurate segmentation results, (**d**) an example of an artifact due to the absence of contact between the US probe and the tissue surface on the left side of the image, (**e**) example of a hydromarker on the right side of the image and an artifact due to acoustic shadowing beneath the tumor lesion. Blue shade: ground truth based human expert annotations; Red shade: automatic tumor segmentation using the optimized weighted average ensemble learning method; Purple shade: the agreement between the human observer and automatic prediction.

Figure 8 displays a few examples of tumor segmentation on BCS-US images using the optimal weighted average ensemble technique. The first three rows in this figure display common examples of specimen US images of malignant lesions with various hyperechogenicity, which were all segmented quite accurately with high DSC scores. However, the last two rows show examples of more complex BCS-US images of malignant tumors, with less accurate segmentation results. The fourth row shows an example of an US image without complete contact between the US probe and the tissue on the left side of the image. This causes a segmentation error, leading to a $TMD_{Pred}$ that is shorter than the $TMD_{Obs}$. The last row shows an example of an US image of a large, malignant lesion accompanied by a hydrogel biopsy site marker on the right side. The marker gets erroneously segmented as a tumor lesion, leading to a lower DSC score and an inaccurate $TMD_{Pred}$. Also, the lower boundaries of the tumor were not correctly segmented due to the acoustic shadowing of the hydrogel.

## 4. Discussion

There is an unmet clinical need for an accurate, fast and efficient adjunctive tool for intraoperative margin assessment during breast-conserving surgery. A promising approach might be the use of ultrasound However, one significant problem is the difficult interpretation of US images due to a low signal-to-noise ratio, artifacts, and the need for experience. Our proposed solution is the use of artificial intelligence-based US evaluation methods. For this purpose, we have introduced a new deep learning framework in order to combine and optimize the segmentation performance and margin assessment performance of 8 pre-trained, previously developed artificial neural network models [47], when applied to a new data set of US images acquired on BCS specimens.

Our first goal was to evaluate the segmentation performance of the 8 individual neural network models on an independent BCS-US data set acquired in our hospital. The highest segmentation performance obtained by these pre-trained individual models was a median DSC score of 0.75 on this data set. In comparison, the original study by Gómez-Flores et al. reported that the highest median DSC when tested on their data set of BUS images of malignant lesions, was 0.88. In fact, none of the individual pre-trained models could achieve the performances reported in the original study, and the median value of the DSCs of all networks is 21% less compared to the median value of the originally reported scores (0.68 versus 0.87). The difference in the performances can be explained by the fact that the models were originally trained on 1) images acquired with US devices using different transducer characteristics or settings, 2) images captured from the outside of the breast, with more contextual tissue information. In our BCS-US data set, a high-resolution transducer was used and the US images were acquired directly on the surface of the lumpectomy specimens. The individual networks seem to lack the robustness to adapt to these types of variations in US images.

In order to mitigate the aforementioned problem, we have introduced a new framework for the purpose of tumor segmentation, combining multiple pre-trained networks using various ensemble approaches. There was an increasing trend in the segmentation performance when switching from general voting to elective voting, to the weighted average technique. The most optimum model combination was obtained using an exhaustive grid search algorithm (DSC 0.88, IQR 0.18). Further investigation showed that the highest segmentation performance comprised a threshold of 0.20 or 0.22, and the highest weights were assigned to all DeepLabV3+ models, except for ResNet18, which had the lowest mean weight factor. These results shed light on the architectures that contributed the most to an accurate segmentation result, even though all mean weight factors were quite close to each other (range 0.08–0.17). Using the most optimum model combination, a median DSC of 0.88 was attained on the BCS-US data set, which was similar to the value mentioned by Gómez-Flores et al. [47] on the BUS data set. This suggests that the use of an ensemble approach is capable of compensating for individual pre-trained models’ lower performance, which enables the application of these models on a wider variety of US images.

Furthermore, we optimized the ensemble approach in order to achieve the highest margin assessment performance. We found that the most optimum NTWC for this goal comprised a threshold of 0.22, and the highest weights were again assigned to all DeepLabV3+ architectures, this time also including the Resnet18 model. It is noteworthy to mention that when it comes to accurate margin assessment, the contribution of DeepLabV3+ architectures is even more important since the mean weight factors of these networks among the best-performing NTWCs were much higher compared to the other networks (range 0.01–0.22). It seems that these networks perform better at detecting the upper boundaries of the tumor, while other networks seem to be only accurate at segmenting the lesion as a whole. Therefore, when using this ensemble approach for other data sets, it is important to be aware that the optimum weight distribution for a high segmentation performance might not be equal to the optimum weight distribution for a high margin assessment performance.

The $TMD_{Pred}$ of the optimum NTWC was compared to two types of ground truth data ($TMD_{HE}$ and $TMD_{Obs}$). When comparing the $TMD_{Pred}$ to the $TMD_{HE}$, the sensitivity for predicting a 2 mm-margin was 96%, the specificity was 76%, the NRMSE was 0.16, and the MAE was 0.57 mm. When looking further at the distribution of the errors in Figure 7a, there was a small mean bias of −0.15 mm (95% CI: −1.68, 1.37) and there was no significant difference between the means of both TMDs. On the other hand, when comparing the $TMD_{Pred}$ to the $TMD_{Obs}$, all calculated margin errors were substantially higher. Similarly, there was a substantial mean bias of −0.56 mm (95% CI: −2.22, 1.10), and the means of both TMDs were significantly different at the 5% level (*p*-value 0.0008) (Figure 7c). This demonstrates that the neural networks are more accurate at predicting the $TMD_{HE}$ than predicting the $TMD_{Obs}$, which is more valuable, since the margin status is eventually determined by the outcome of the histopathological examination.

In addition, there was a significant difference between the means of $TMD_{Obs}$ and $TMD_{HE}$ (*p*-value 0.0090). The sensitivity of the human expert for predicting a close margin in the H&E section was 87%, and the specificity was 82%. The lower specificity of the $TMD_{Pred}$ (76%) and the $TMD_{Obs}$ (82%) when it comes to predicting the $TMD_{HE}$, might be due to human error in accurately marking the tumor margin as well as tissue deformation caused by pathology processing. It is worthwile to note that the $TMD_{Pred}$ and the $TMD_{Obs}$ could both be affected by variable tissue compression due to ultrasound probe pressure. This effect is user-dependent and has not been investigated in this study. However, we expect this effect to be quite minimal since lumpectomy specimens are smaller and stiffer (due to the tumor inside) than the breast. It was evident that the optimized ensemble approach was quite more accurate at estimating the $TMD_{HE}$, and had a higher sensitivity for predicting a close margin compared to the human observed tumor-margin distance. This demonstrates the potential value of BCS-US segmentation based on an ensemble approach for margin assessment.

In current surgical practice, several techniques are used for intra-operative resection margin assessment. These techniques include specimen radiography, intraoperative frozen section analysis, and intraoperative touch preparation cytology. In a meta-analysis by Chen Lin et al. based on 20 different studies, it was found that 2D specimen mammography has a pooled weighted sensitivity of 55%, and a pooled weighted specificity of 85% for detecting a positive resection margin [48]. Furthermore, in a systematic review of Esbona et al. based on 41 different patient cohorts, it was found that imprint cytology and frozen section analysis has a pooled sensitivity of 72% respectively 83%, and a pooled specificity of 97% respectively 95% [49]. Our proposed margin assessment method has a higher sensitivity (96%) compared to all of these currently used techniques, which is highly essential for a better patient outcome. Furthermore, our proposed method mitigates logistical issues that hamper broad acceptance, including the complexity, and time-consuming nature of the currently used techniques, and the workload for pathologists and/or radiologists. Besides our proposed method, many novel techniques are being investigated to support margin assessment, including fluorescence imaging [50], Raman spectroscopy [51,52], optical coherence tomography (OCT) [53,54,55], radiofrequency (RF) spectroscopy [56,57], bioimpedance spectroscopy [58], micro-computed tomography (micro-CT) [59], digital breast tomosynthesis [60], ultraviolet-photoacoustic microscopy (UV-PAM) [61], microscopy with ultraviolet surface excitation (MUSE) and photoacoustic tomography [62]. However, these techniques have not been included yet in the surgical workflow due to various reasons. Some techniques have low diagnostic accuracy, including digital breast tomosynthesis (sensitivity of 74%) [60] and RF spectroscopy (sensitivity of 71%) [63], micro-CT (sensitivity 56%) [59]. Other techniques are too time-consuming, such as ultraviolet-photoacoustic microscopy (UV-PAM) of which the analysis could take up several hours. A rapid method for margin assessment is MUSE, which also seems cost-effective [64]. However, the authors report a sensitivity of 88% which is lower compared to the sensitivity of CNN-based US evaluation for margin assessment (96%) [64]. Other techniques including fluorescence spectroscopy, Raman spectroscopy and bioimpedance spectroscopy have promising results, but the cost-effectiveness and operating speed have not been investigated yet [65,66,67,68]. In contrast to downsides of other novel techniques, artificial intelligence-based US evaluation seems to be a promising adjunctive tool for intraoperative margin assessment that fits seamlessly within the surgical workflow.

An important remark is that the growth patterns of breast cancer lesions are highly different and depend on the histological tumor type, grade and immunohistochemically defined subtype. The treatment plan including the surgical steps is based on the aforementioned lesion characteristics. For this study, we have not included enough patients to determine the margin assessment performance for different lesion characteristics. However, this would be a valuable step for future investigations as described below.

In order to make this technology even more valuable in the future, the used algorithms could be improved further. Therefore, we are planning to acquire more annotated BCS-US data, which will allow applying transfer learning to improve segmentation performance. Additionally, in order to correcly distinguish tumorous lesions from tissue markers and hematomas, more US images displaying these artifacts should be acquired and used as training input for the algorithms. Furthermore, US images from healthy breast tissue need to be acquired for training, since the algorithms were only trained on images with tumor lesions. Additionally, the level of tissue deformation induced by ultrasound pressure, could be investigated. Furthermore, the ultrasound device settings including gain, time gain compensation and focus should be optimized in order to improve the quality of the images further. A further prospective clinical in vivo study needs to be conducted to assess the sensitivity, specificity and accuracy of this new method for real-time predicting a close margin. Furthermore, after collecting a larger dataset, subanalyses could be performed to determine the performance values for different histological tumor characteristics (e.g., tumor type, grade and level of invasiveness), and biological tumor characteristics (e.g., subtypes based on hormonal receptors. The other purpose of this study would be to evaluate the implementation of this method in the clinical work routine.

## 5. Conclusions

In this paper, we have developed and evaluated new ensemble approaches for automated tumor segmentation in BCS-US images based on 8 pre-trained, public deep learning models, for the purpose of predicting close margins (≤2.0 mm), in US images of breast cancer specimens. The most optimum ensemble approach for segmentation yielded a median DSC of 0.88 on our data set. On the other hand, the most optimum ensemble approach for margin assessment yielded a sensitivity of 96% and a specificity of 76% for predicting a close margin (≤2.0 mm) in the H&E section. These results show the potential of the proposed method for margin assessment. Additional data acquisition to improve the algorithms, and a large clinical in vivo study to evaluate this new method would be the next steps towards reaching the ultimate goal of intraoperative margin assessment using automated BUS segmentation.

## Figures and Tables

**Table 1:** Tumor and patient characteristics.

	Characteristic N = 86
**Age** (years) (median, SD)	57 (12.4)
**Menopausal status**	
Pre	26 (30%)
Post	60 (70%)
**BMI** (kg/m2) (median, SD)	25 (4.1)
**Lesion diameter** (mm) (median, min, max)	1.5 (0.4, 5.5)
**Specimen weight** (gram) (median, SD)	20 (19)
**Histological tumor type**	
IC NST	35 (41%)
IC NST + DCIS	43 (50%)
ILC	3 (3%)
ILC + LCIS	5 (6%)
**T-stage**	
pT1a	8 (9%)
pT1b	16 (19%)
pT1c	38 (44%)
pT2	23 (27%)
pT3	1 (1%)
**Histological tumor grade**	
1	36 (42%)
2	44 (51%)
3	6 (7%)
**Hormonal receptor status**	
ER+	81 (94%)
ER-	5 (6%)
PR+	60 (70%)
PR-	26 (30%)
**HER2 status**	
HER2+	8 (9%)
HER2-	78 (91%)
**Immunohistochemically defined subtype**	
Luminal A-like	54 (63%)
Luminal B-like/HER2-negative	22 (26%)
Luminal B-like/HER2-positive	7 (8%)
HER2-positive	1 (1%)
TNBC	2 (2%)
Neoadjuvant treatment	
Chemotherapy +/− targeted therapy	16 (19%)
Endocrine therapy	14 (16%)
None	56 (65%)

IC NST = invasive carcinoma of no special type, DCIS = ductal carcinoma in situ; ER = estrogen receptor, PR = progesterone receptor; Luminal A-like (ER- and/or PR-positive, HER2-negative, Ki-67 < 20%); Luminal B-like/HER2-negative (ER- and/or PR-positive/HER2-negative/Ki-67 ≥ 20%); Luminal B-like/HER2-positive (ER- and/or PR-positive/HER2-positive/any Ki-67 value); HER2-positive (ER- and PR-negative/HER2-positive); TNBC = triple negative breast cancer (ER- and PR-negative/HER2-negative).

**Table 2:** Selected weights and thresholds after parameter optimization.

	AlexNet	MobileNet	ResNet18	ResNet50	U-Net	VGG16	VGG19	Xception	Threshold
**Maximizing DSC**	0.04	0.25	0	0.18	0.11	0.07	0.14	0.21	0.22
**Maximizing TDM sensitivity**	0.11	0.14	0.21	0.25	0.04	0	0.07	0.18	0.22

**Table 3:** Median DSC of the final segmentation method on three different randomly selected subsets.

	Median DSC (IQR)
**Data subset 1**	0.86 (0.15)
**Data subset 2**	0.85 (0.19)
**Data subset 3**	0.89 (0.19)

**Table 4:** Tumor margin distance errors, margin assessment performance, and *p*-values of two-sample *t*-tests for different comparisons of TMDs.

	TMD Error			Margin Assessment Performance		*t*-Test
	MAE (mm)	NRMSE	PCC	Sensitivity	Specificity	*p*-Value
$TMD_{Pred}$ **vs.** $TMD_{HE}$	**0.57**	**0.16**	**0.72**	**96%**	76%	**0.3736**
$TMD_{Pred}$ **vs.** $TMD_{obs}$	0.83	0.21	0.70	95%	57%	0.0008 *
$TMD_{obs}$ **vs.** $TMD_{HE}$	0.73	0.19	0.69	87%	**82%**	0.0090 *

* indicate a significant difference (*p*-value ≤ 0.05); TMDPred: automatic predicted tumor margin; *TMD_HE_*: tumor margin based on histology results; *TMD_Obs_*: extracted tumor margin by an expert ultrasound device annotation tool.

## Data Availability

Data underlying the results presented in this paper are not publicly available at time but may be obtained from the authors upon reasonable request.
